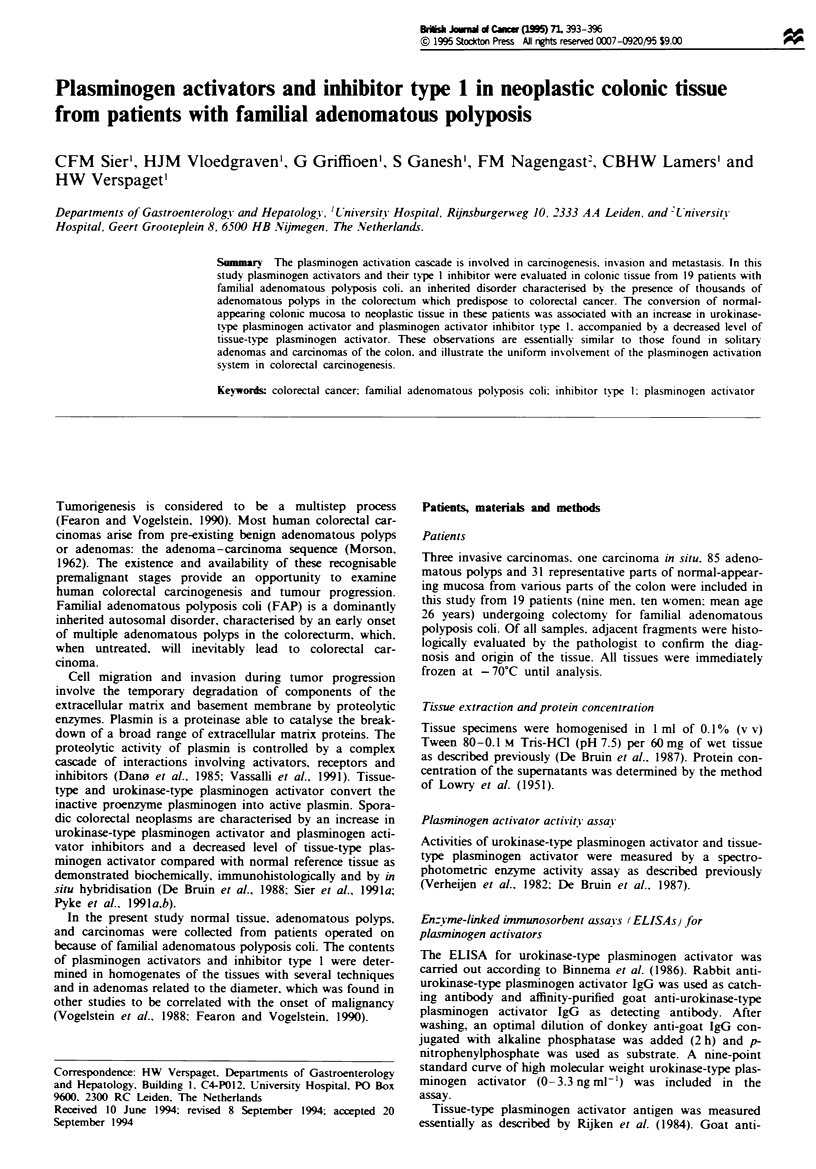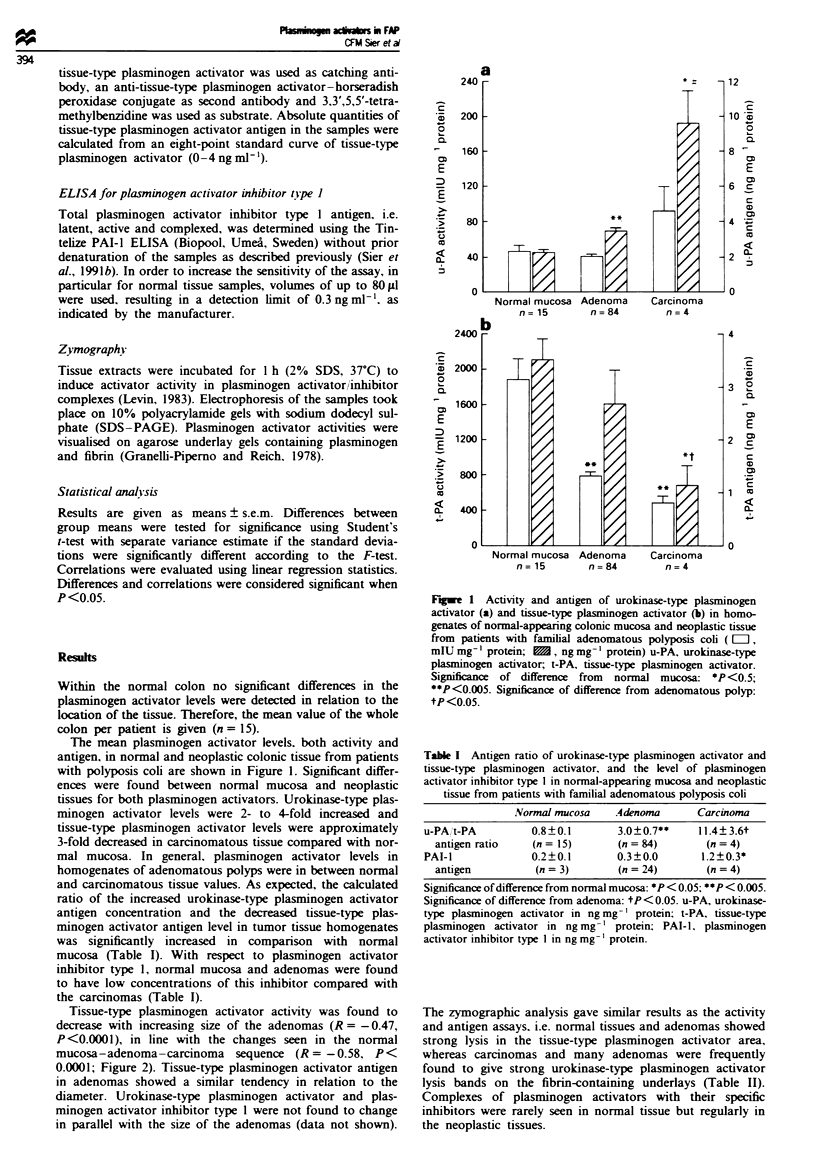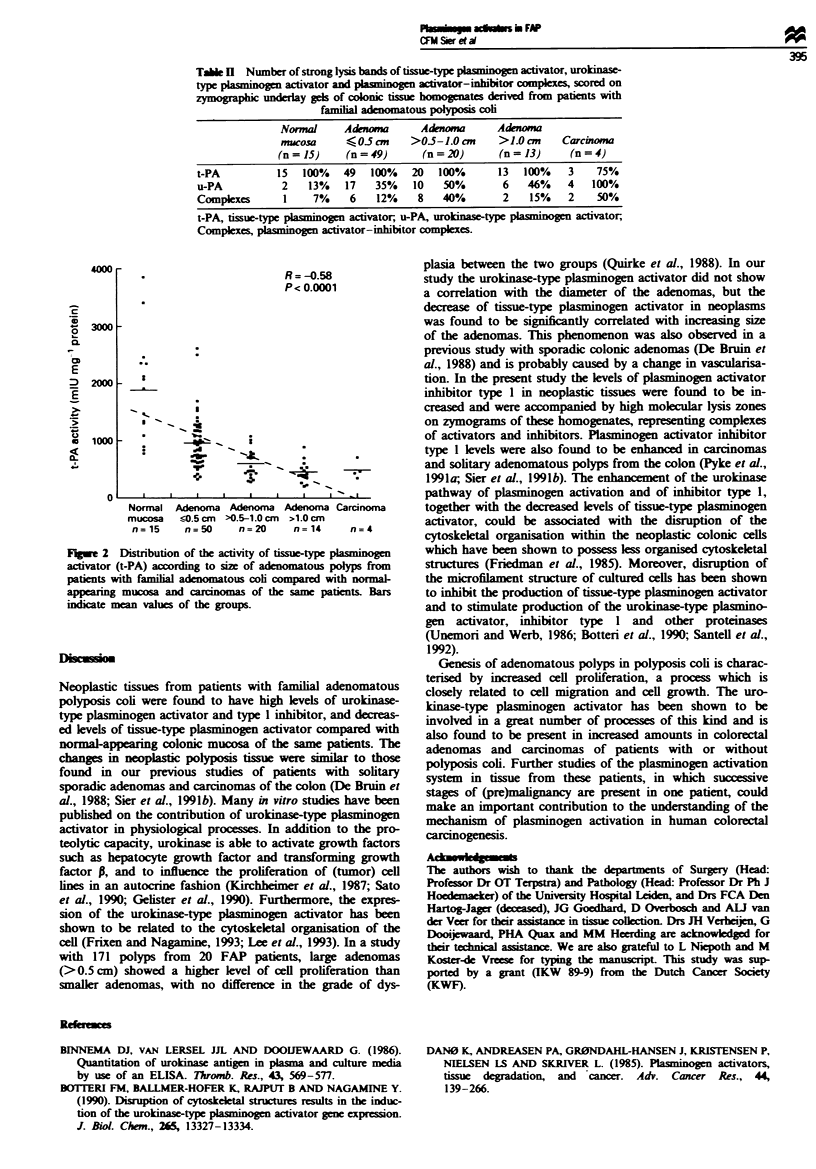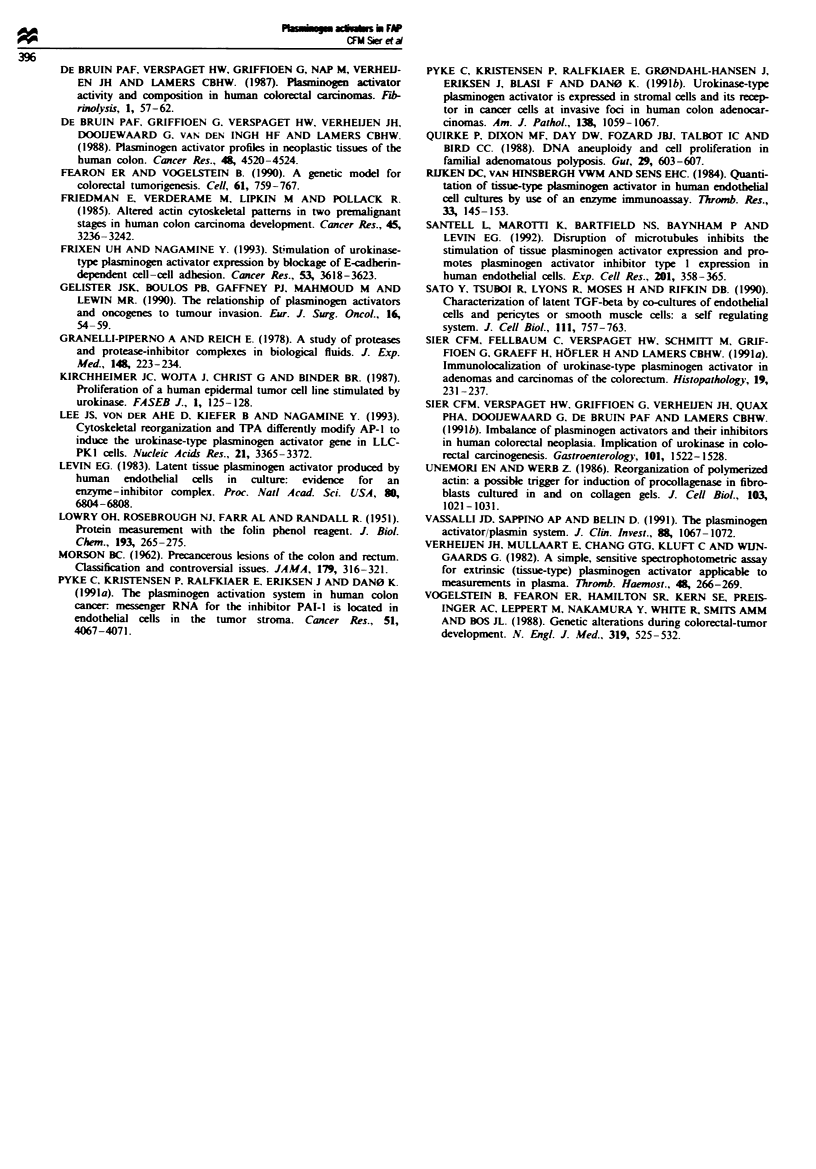# Plasminogen activators and inhibitor type 1 in neoplastic colonic tissue from patients with familial adenomatous polyposis.

**DOI:** 10.1038/bjc.1995.80

**Published:** 1995-02

**Authors:** C. F. Sier, H. J. Vloedgraven, G. Griffioen, S. Ganesh, F. M. Nagengast, C. B. Lamers, H. W. Verspaget

**Affiliations:** Department of Gastroenterology and Hepatology, University Hospital, Leiden, The Netherlands.

## Abstract

The plasminogen activation cascade is involved in carcinogenesis, invasion and metastasis. In this study plasminogen activators and their type 1 inhibitor were evaluated in colonic tissue from 19 patients with familial adenomatous polyposis coli, an inherited disorder characterised by the presence of thousands of adenomatous polyps in the colorectum which predispose to colorectal cancer. The conversion of normal-appearing colonic mucosa to neoplastic tissue in these patients was associated with an increase in urokinase-type plasminogen activator and plasminogen activator inhibitor type 1, accompanied by a decreased level of tissue-type plasminogen activator. These observations are essentially similar to those found in solitary adenomas and carcinomas of the colon, and illustrate the uniform involvement of the plasminogen activation system in colorectal carcinogenesis.


British Journal of Cancer (1995) 71, 393-396

© 1995 Stockton Press All rights reserved 0007-0920/95 $9.00

### Plasminogen activators and inhibitor type 1 in neoplastic colonic tissue from patients with familial adenomatous polyposis

CFM Sier[1], HJM Vloedgraven[1], G Griffioen[1], S Ganesh[1], FM Nagengast[2], CBHW Lamers[1] and HW Verspaget[1]

*Departments of Gastroenterology and Hepatology, [1]University Hospital, Rijnsburgerweg 10, 2333 AA Leiden, and [2]University Hospital, Geert Grooteplein 8, 6500 HB Nijmegen, The Netherlands.*

**Summary** The plasminogen activation cascade is involved in carcinogenesis, invasion and metastasis. In this study plasminogen activators and their type 1 inhibitor were evaluated in colonic tissue from 19 patients with familial adenomatous polyposis coli, an inherited disorder characterised by the presence of thousands of adenomatous polyps in the colorectum which predispose to colorectal cancer. The conversion of normal-appearing colonic mucosa to neoplastic tissue in these patients was associated with an increase in urokinase-type plasminogen activator and plasminogen activator inhibitor type 1, accompanied by a decreased level of tissue-type plasminogen activator. These observations are essentially similar to those found in solitary adenomas and carcinomas of the colon, and illustrate the uniform involvement of the plasminogen activation system in colorectal carcinogenesis.

**Keywords:** colorectal cancer; familial adenomatous polyposis coli; inhibitor type 1; plasminogen activator

Tumorigenesis is considered to be a multistep process (Fearon and Vogelstein, 1990). Most human colorectal carcinomas arise from pre-existing benign adenomatous polyps or adenomas: the adenoma–carcinoma sequence (Morson, 1962). The existence and availability of these recognisable premalignant stages provide an opportunity to examine human colorectal carcinogenesis and tumour progression. Familial adenomatous polyposis coli (FAP) is a dominantly inherited autosomal disorder, characterised by an early onset of multiple adenomatous polyps in the colorecturm, which, when untreated, will inevitably lead to colorectal carcinoma.

Cell migration and invasion during tumor progression involve the temporary degradation of components of the extracellular matrix and basement membrane by proteolytic enzymes. Plasmin is a proteinase able to catalyse the breakdown of a broad range of extracellular matrix proteins. The proteolytic activity of plasmin is controlled by a complex cascade of interactions involving activators, receptors and inhibitors (Danø *et al.*, 1985; Vassalli *et al.*, 1991). Tissue-type and urokinase-type plasminogen activator convert the inactive proenzyme plasminogen into active plasmin. Sporadic colorectal neoplasms are characterised by an increase in urokinase-type plasminogen activator and plasminogen activator inhibitors and a decreased level of tissue-type plasminogen activator compared with normal reference tissue as demonstrated biochemically, immunohistologically and by *in situ* hybridisation (De Bruin *et al.*, 1988; Sier *et al.*, 1991*a*; Pyke *et al.*, 1991*a,b*).

In the present study normal tissue, adenomatous polyps, and carcinomas were collected from patients operated on because of familial adenomatous polyposis coli. The contents of plasminogen activators and inhibitor type 1 were determined in homogenates of the tissues with several techniques and in adenomas related to the diameter, which was found in other studies to be correlated with the onset of malignancy (Vogelstein *et al.*, 1988; Fearon and Vogelstein, 1990).

Correspondence: HW Verspaget, Departments of Gastroenterology and Hepatology, Building 1, C4-P012, University Hospital, PO Box 9600, 2300 RC Leiden, The Netherlands
Received 10 June 1994; revised 8 September 1994; accepted 20 September 1994

### Patients, materials and methods

### *Patients*

Three invasive carcinomas, one carcinoma *in situ*, 85 adenomatous polyps and 31 representative parts of normal-appearing mucosa from various parts of the colon were included in this study from 19 patients (nine men, ten women; mean age 26 years) undergoing colectomy for familial adenomatous polyposis coli. Of all samples, adjacent fragments were histologically evaluated by the pathologist to confirm the diagnosis and origin of the tissue. All tissues were immediately frozen at −70°C until analysis.

### *Tissue extraction and protein concentration*

Tissue specimens were homogenised in 1 ml of 0.1% (v/v) Tween 80–0.1 M Tris-HCl (pH 7.5) per 60 mg of wet tissue as described previously (De Bruin *et al.*, 1987). Protein concentration of the supernatants was determined by the method of Lowry *et al.* (1951).

### *Plasminogen activator activity assay*

Activities of urokinase-type plasminogen activator and tissue-type plasminogen activator were measured by a spectrophotometric enzyme activity assay as described previously (Verheijen *et al.*, 1982; De Bruin *et al.*, 1987).

### *Enzyme-linked immunosorbent assays (ELISAs) for plasminogen activators*

The ELISA for urokinase-type plasminogen activator was carried out according to Binnema *et al.* (1986). Rabbit anti-urokinase-type plasminogen activator IgG was used as catching antibody and affinity-purified goat anti-urokinase-type plasminogen activator IgG as detecting antibody. After washing, an optimal dilution of donkey anti-goat IgG conjugated with alkaline phosphatase was added (2 h) and *p*-nitrophenylphosphate was used as substrate. A nine-point standard curve of high molecular weight urokinase-type plasminogen activator (0–3.3 ng ml$^{-1}$) was included in the assay.

Tissue-type plasminogen activator antigen was measured essentially as described by Rijken *et al.* (1984). Goat antitissue-type plasminogen activator was used as catching antibody, an anti-tissue-type plasminogen activator–horseradish peroxidase conjugate as second antibody and 3,3',5,5'-tetramethylbenzidine was used as substrate. Absolute quantities of tissue-type plasminogen activator antigen in the samples were calculated from an eight-point standard curve of tissue-type plasminogen activator (0–4 ng $ml^{-1}$).

### *ELISA for plasminogen activator inhibitor type 1*

Total plasminogen activator inhibitor type 1 antigen, i.e. latent, active and complexed, was determined using the Tintelize PAI-1 ELISA (Biopool, Umeå, Sweden) without prior denaturation of the samples as described previously (Sier *et al.*, 1991*b*). In order to increase the sensitivity of the assay, in particular for normal tissue samples, volumes of up to 80 µl were used, resulting in a detection limit of 0.3 ng $ml^{-1}$, as indicated by the manufacturer.

### *Zymography*

Tissue extracts were incubated for 1 h (2% SDS, 37°C) to induce activator activity in plasminogen activator/inhibitor complexes (Levin, 1983). Electrophoresis of the samples took place on 10% polyacrylamide gels with sodium dodecyl sulphate (SDS–PAGE). Plasminogen activator activities were visualised on agarose underlay gels containing plasminogen and fibrin (Granelli-Piperno and Reich, 1978).

### *Statistical analysis*

Results are given as means ± s.e.m. Differences between group means were tested for significance using Student's *t*-test with separate variance estimate if the standard deviations were significantly different according to the *F*-test. Correlations were evaluated using linear regression statistics. Differences and correlations were considered significant when $P<0.05$.

### Results

Within the normal colon no significant differences in the plasminogen activator levels were detected in relation to the location of the tissue. Therefore, the mean value of the whole colon per patient is given ($n = 15$).

The mean plasminogen activator levels, both activity and antigen, in normal and neoplastic colonic tissue from patients with polyposis coli are shown in Figure 1. Significant differences were found between normal mucosa and neoplastic tissues for both plasminogen activators. Urokinase-type plasminogen activator levels were 2- to 4-fold increased and tissue-type plasminogen activator levels were approximately 3-fold decreased in carcinomatous tissue compared with normal mucosa. In general, plasminogen activator levels in homogenates of adenomatous polyps were in between normal and carcinomatous tissue values. As expected, the calculated ratio of the increased urokinase-type plasminogen activator antigen concentration and the decreased tissue-type plasminogen activator antigen level in tumor tissue homogenates was significantly increased in comparison with normal mucosa (Table I). With respect to plasminogen activator inhibitor type 1, normal mucosa and adenomas were found to have low concentrations of this inhibitor compared with the carcinomas (Table I).

Tissue-type plasminogen activator activity was found to decrease with increasing size of the adenomas ($R = -0.47$, $P<0.0001$), in line with the changes seen in the normal mucosa–adenoma–carcinoma sequence ($R = -0.58$, $P<0.0001$; Figure 2). Tissue-type plasminogen activator antigen in adenomas showed a similar tendency in relation to the diameter. Urokinase-type plasminogen activator and plasminogen activator inhibitor type 1 were not found to change in parallel with the size of the adenomas (data not shown).

**Figure 1** Activity and antigen of urokinase-type plasminogen activator (**a**) and tissue-type plasminogen activator (**b**) in homogenates of normal-appearing colonic mucosa and neoplastic tissue from patients with familial adenomatous polyposis coli (□, mIU $mg^{-1}$ protein; ▨, ng $mg^{-1}$ protein) u-PA, urokinase-type plasminogen activator; t-PA, tissue-type plasminogen activator. Significance of difference from normal mucosa: $^{*}P<0.5$; $^{**}P<0.005$. Significance of difference from adenomatous polyp: $^{\dagger}P<0.05$.

**Table I** Antigen ratio of urokinase-type plasminogen activator and tissue-type plasminogen activator, and the level of plasminogen activator inhibitor type 1 in normal-appearing mucosa and neoplastic tissue from patients with familial adenomatous polyposis coli

| | *Normal mucosa* | *Adenoma* | *Carcinoma* |
|---|---|---|---|
| u-PA/t-PA | 0.8±0.1 | 3.0±0.7** | 11.4±3.6† |
| antigen ratio | ($n$ = 15) | ($n$ = 84) | ($n$ = 4) |
| PAI-1 | 0.2±0.1 | 0.3±0.0 | 1.2±0.3* |
| antigen | ($n$ = 3) | ($n$ = 24) | ($n$ = 4) |

Significance of difference from normal mucosa: $^{*}P<0.05$; $^{**}P<0.005$. Significance of difference from adenoma: $^{\dagger}P<0.05$. u-PA, urokinase-type plasminogen activator in ng $mg^{-1}$ protein; t-PA, tissue-type plasminogen activator in ng $mg^{-1}$ protein; PAI-1, plasminogen activator inhibitor type 1 in ng $mg^{-1}$ protein.

The zymographic analysis gave similar results as the activity and antigen assays, i.e. normal tissues and adenomas showed strong lysis in the tissue-type plasminogen activator area, whereas carcinomas and many adenomas were frequently found to give strong urokinase-type plasminogen activator lysis bands on the fibrin-containing underlays (Table II). Complexes of plasminogen activators with their specific inhibitors were rarely seen in normal tissue but regularly in the neoplastic tissues.

**Table II** Number of strong lysis bands of tissue-type plasminogen activator, urokinase-type plasminogen activator and plasminogen activator–inhibitor complexes, scored on zymographic underlay gels of colonic tissue homogenates derived from patients with familial adenomatous polyposis coli

| | *Normal mucosa* (n = *15*) | | *Adenoma* ⩽*0.5 cm* (n = *49*) | | *Adenoma* >*0.5–1.0 cm* (n = *20*) | | *Adenoma* >*1.0 cm* (n = *13*) | | *Carcinoma* (n = *4*) | |
|---|---|---|---|---|---|---|---|---|---|---|
| t-PA | 15 | 100% | 49 | 100% | 20 | 100% | 13 | 100% | 3 | 75% |
| u-PA | 2 | 13% | 17 | 35% | 10 | 50% | 6 | 46% | 4 | 100% |
| Complexes | 1 | 7% | 6 | 12% | 8 | 40% | 2 | 15% | 2 | 50% |

t-PA, tissue-type plasminogen activator; u-PA, urokinase-type plasminogen activator; Complexes, plasminogen activator–inhibitor complexes.

**Figure 2** Distribution of the activity of tissue-type plasminogen activator (t-PA) according to size of adenomatous polyps from patients with familial adenomatous coli compared with normal-appearing mucosa and carcinomas of the same patients. Bars indicate mean values of the groups.

### Discussion

Neoplastic tissues from patients with familial adenomatous polyposis coli were found to have high levels of urokinase-type plasminogen activator and type 1 inhibitor, and decreased levels of tissue-type plasminogen activator compared with normal-appearing colonic mucosa of the same patients. The changes in neoplastic polyposis tissue were similar to those found in our previous studies of patients with solitary sporadic adenomas and carcinomas of the colon (De Bruin *et al.*, 1988; Sier *et al.*, 1991*b*). Many *in vitro* studies have been published on the contribution of urokinase-type plasminogen activator in physiological processes. In addition to the proteolytic capacity, urokinase is able to activate growth factors such as hepatocyte growth factor and transforming growth factor $\beta$, and to influence the proliferation of (tumor) cell lines in an autocrine fashion (Kirchheimer *et al.*, 1987; Sato *et al.*, 1990; Gelister *et al.*, 1990). Furthermore, the expression of the urokinase-type plasminogen activator has been shown to be related to the cytoskeletal organisation of the cell (Frixen and Nagamine, 1993; Lee *et al.*, 1993). In a study with 171 polyps from 20 FAP patients, large adenomas (>0.5 cm) showed a higher level of cell proliferation than smaller adenomas, with no difference in the grade of dysplasia between the two groups (Quirke *et al.*, 1988). In our study the urokinase-type plasminogen activator did not show a correlation with the diameter of the adenomas, but the decrease of tissue-type plasminogen activator in neoplasms was found to be significantly correlated with increasing size of the adenomas. This phenomenon was also observed in a previous study with sporadic colonic adenomas (De Bruin *et al.*, 1988) and is probably caused by a change in vascularisation. In the present study the levels of plasminogen activator inhibitor type 1 in neoplastic tissues were found to be increased and were accompanied by high molecular lysis zones on zymograms of these homogenates, representing complexes of activators and inhibitors. Plasminogen activator inhibitor type 1 levels were also found to be enhanced in carcinomas and solitary adenomatous polyps from the colon (Pyke *et al.*, 1991*a*; Sier *et al.*, 1991*b*). The enhancement of the urokinase pathway of plasminogen activation and of inhibitor type 1, together with the decreased levels of tissue-type plasminogen activator, could be associated with the disruption of the cytoskeletal organisation within the neoplastic colonic cells which have been shown to possess less organised cytoskeletal structures (Friedman *et al.*, 1985). Moreover, disruption of the microfilament structure of cultured cells has been shown to inhibit the production of tissue-type plasminogen activator and to stimulate production of the urokinase-type plasminogen activator, inhibitor type 1 and other proteinases (Unemori and Werb, 1986; Botteri *et al.*, 1990; Santell *et al.*, 1992).

Genesis of adenomatous polyps in polyposis coli is characterised by increased cell proliferation, a process which is closely related to cell migration and cell growth. The urokinase-type plasminogen activator has been shown to be involved in a great number of processes of this kind and is also found to be present in increased amounts in colorectal adenomas and carcinomas of patients with or without polyposis coli. Further studies of the plasminogen activation system in tissue from these patients, in which successive stages of (pre)malignancy are present in one patient, could make an important contribution to the understanding of the mechanism of plasminogen activation in human colorectal carcinogenesis.

**Acknowledgements**

The authors wish to thank the departments of Surgery (Head: Professor Dr OT Terpstra) and Pathology (Head: Professor Dr Ph J Hoedemaeker) of the University Hospital Leiden, and Drs FCA Den Hartog-Jager (deceased), JG Goedhard, D Overbosch and ALJ van der Veer for their assistance in tissue collection. Drs JH Verheijen, G Dooijewaard, PHA Quax and MM Heerding are acknowledged for their technical assistance. We are also grateful to L Niepoth and M Koster-de Vreese for typing the manuscript. This study was supported by a grant (IKW 89-9) from the Dutch Cancer Society (KWF).

### References

BINNEMA DJ, VAN LERSEL JJL AND DOOIJEWAARD G. (1986). Quantitation of urokinase antigen in plasma and culture media by use of an ELISA. *Thromb. Res.*, **43**, 569–577.

BOTTERI FM, BALLMER-HOFER K, RAJPUT B AND NAGAMINE Y. (1990). Disruption of cytoskeletal structures results in the induction of the urokinase-type plasminogen activator gene expression. *J. Biol. Chem.*, **265**, 13327–13334.

DANØ K, ANDREASEN PA, GRØNDAHL-HANSEN J, KRISTENSEN P, NIELSEN LS AND SKRIVER L. (1985). Plasminogen activators, tissue degradation, and cancer. *Adv. Cancer Res.*, **44**, 139–266.

De Bruin PAF, Verspaget HW, Griffioen G, Nap M, Verheijen JH and Lamers CBHW. (1987). Plasminogen activator activity and composition in human colorectal carcinomas. *Fibrinolysis*, **1**, 57–62.

De Bruin PAF, Griffioen G, Verspaget HW, Verheijen JH, Dooijewaard G, van den Ingh HF and Lamers CBHW. (1988). Plasminogen activator profiles in neoplastic tissues of the human colon. *Cancer Res.*, **48**, 4520–4524.

Fearon ER and Vogelstein B. (1990). A genetic model for colorectal tumorigenesis. *Cell*, **61**, 759–767.

Friedman E, Verderame M, Lipkin M and Pollack R. (1985). Altered actin cytoskeletal patterns in two premalignant stages in human colon carcinoma development. *Cancer Res.*, **45**, 3236–3242.

Frixen UH and Nagamine Y. (1993). Stimulation of urokinase-type plasminogen activator expression by blockage of E-cadherin-dependent cell–cell adhesion. *Cancer Res.*, **53**, 3618–3623.

Gelister JSK, Boulos PB, Gaffney PJ, Mahmoud M and Lewin MR. (1990). The relationship of plasminogen activators and oncogenes to tumour invasion. *Eur. J. Surg. Oncol.*, **16**, 54–59.

Granelli-Piperno A and Reich E. (1978). A study of proteases and protease-inhibitor complexes in biological fluids. *J. Exp. Med.*, **148**, 223–234.

Kirchheimer JC, Wojta J, Christ G and Binder BR. (1987). Proliferation of a human epidermal tumor cell line stimulated by urokinase. *FASEB J.*, **1**, 125–128.

Lee JS, von der Ahe D, Kiefer B and Nagamine Y. (1993). Cytoskeletal reorganization and TPA differently modify AP-1 to induce the urokinase-type plasminogen activator gene in LLC-PK1 cells. *Nucleic Acids Res.*, **21**, 3365–3372.

Levin EG. (1983). Latent tissue plasminogen activator produced by human endothelial cells in culture: evidence for an enzyme–inhibitor complex. *Proc. Natl Acad. Sci. USA*, **80**, 6804–6808.

Lowry OH, Rosebrough NJ, Farr AL and Randall R. (1951). Protein measurement with the folin phenol reagent. *J. Biol. Chem.*, **193**, 265–275.

Morson BC. (1962). Precancerous lesions of the colon and rectum. Classification and controversial issues. *JAMA*, **179**, 316–321.

Pyke C, Kristensen P, Ralfkiaer E, Eriksen J and Danø K. (1991*a*). The plasminogen activation system in human colon cancer: messenger RNA for the inhibitor PAI-1 is located in endothelial cells in the tumor stroma. *Cancer Res.*, **51**, 4067–4071.

Pyke C, Kristensen P, Ralfkiaer E, Grøndahl-Hansen J, Eriksen J, Blasi F and Danø K. (1991*b*). Urokinase-type plasminogen activator is expressed in stromal cells and its receptor in cancer cells at invasive foci in human colon adenocarcinomas. *Am. J. Pathol.*, **138**, 1059–1067.

Quirke P, Dixon MF, Day DW, Fozard JBJ, Talbot IC and Bird CC. (1988). DNA aneuploidy and cell proliferation in familial adenomatous polyposis. *Gut*, **29**, 603–607.

Rijken DC, van Hinsbergh VWM and Sens EHC. (1984). Quantitation of tissue-type plasminogen activator in human endothelial cell cultures by use of an enzyme immunoassay. *Thromb. Res.*, **33**, 145–153.

Santell L, Marotti K, Bartfield NS, Baynham P and Levin EG. (1992). Disruption of microtubules inhibits the stimulation of tissue plasminogen activator expression and promotes plasminogen activator inhibitor type 1 expression in human endothelial cells. *Exp. Cell Res.*, **201**, 358–365.

Sato Y, Tsuboi R, Lyons R, Moses H and Rifkin DB. (1990). Characterization of latent TGF-beta by co-cultures of endothelial cells and pericytes or smooth muscle cells: a self regulating system. *J. Cell Biol.*, **111**, 757–763.

Sier CFM, Fellbaum C, Verspaget HW, Schmitt M, Griffioen G, Graeff H, Höfler H and Lamers CBHW. (1991*a*). Immunolocalization of urokinase-type plasminogen activator in adenomas and carcinomas of the colorectum. *Histopathology*, **19**, 231–237.

Sier CFM, Verspaget HW, Griffioen G, Verheijen JH, Quax PHA, Dooijewaard G, De Bruin PAF and Lamers CBHW. (1991*b*). Imbalance of plasminogen activators and their inhibitors in human colorectal neoplasia. Implication of urokinase in colorectal carcinogenesis. *Gastroenterology*, **101**, 1522–1528.

Unemori EN and Werb Z. (1986). Reorganization of polymerized actin: a possible trigger for induction of procollagenase in fibroblasts cultured in and on collagen gels. *J. Cell Biol.*, **103**, 1021–1031.

Vassalli JD, Sappino AP and Belin D. (1991). The plasminogen activator/plasmin system. *J. Clin. Invest.*, **88**, 1067–1072.

Verheijen JH, Mullaart E, Chang GTG, Kluft C and Wijngaards G. (1982). A simple, sensitive spectrophotometric assay for extrinsic (tissue-type) plasminogen activator applicable to measurements in plasma. *Thromb. Haemost.*, **48**, 266–269.

Vogelstein B, Fearon ER, Hamilton SR, Kern SE, Preisinger AC, Leppert M, Nakamura Y, White R, Smits AMM and Bos JL. (1988). Genetic alterations during colorectal-tumor development. *N. Engl. J. Med.*, **319**, 525–532.